# Physiological Tolerance Times while Wearing Explosive Ordnance Disposal Protective Clothing in Simulated Environmental Extremes

**DOI:** 10.1371/journal.pone.0083740

**Published:** 2014-02-21

**Authors:** Ian B. Stewart, Kelly L. Stewart, Charles J. Worringham, Joseph T. Costello

**Affiliations:** School of Exercise and Nutrition Sciences and Institute of Health and Biomedical Innovation, Queensland University of Technology, Brisbane, Queensland, Australia; The University of Queensland, Australia

## Abstract

Explosive ordnance disposal (EOD) technicians are required to wear protective clothing to protect themselves from the threat of overpressure, fragmentation, impact and heat. The engineering requirements to minimise these threats results in an extremely heavy and cumbersome clothing ensemble that increases the internal heat generation of the wearer, while the clothing’s thermal properties reduce heat dissipation. This study aimed to evaluate the heat strain encountered wearing EOD protective clothing in simulated environmental extremes across a range of differing work intensities. Eight healthy males [age 25±6 years (mean ± sd), height 180±7 cm, body mass 79±9 kg, V˙O_2max_ 57±6 ml^.^kg^−1.^min^−1^] undertook nine trials while wearing an EOD9 suit (weighing 33.4 kg). The trials involved walking on a treadmill at 2.5, 4 and 5.5 km⋅h^−1^ at each of the following environmental conditions, 21, 30 and 37°C wet bulb globe temperature (WBGT) in a randomised controlled crossover design. The trials were ceased if the participants’ core temperature reached 39°C, if heart rate exceeded 90% of maximum, if walking time reached 60 minutes or due to fatigue/nausea. Tolerance times ranged from 10–60 minutes and were significantly reduced in the higher walking speeds and environmental conditions. In a total of 15 trials (21%) participants completed 60 minutes of walking; however, this was predominantly at the slower walking speeds in the 21°C WBGT environment. Of the remaining 57 trials, 50 were ceased, due to attainment of 90% maximal heart rate. These near maximal heart rates resulted in moderate-high levels of physiological strain in all trials, despite core temperature only reaching 39°C in one of the 72 trials.

## Introduction

Injury and deaths attributed to improvised explosive devices (IED) have exponentially increased in the last ten years [1]. While the use of IEDs is not unique to modern warfare, they have become the weapon of choice for terrorist and guerrilla attacks on civilian and army personnel. This has led to an increased role for explosive ordnance disposal (EOD) technicians in the search, disarmament and clearance of IEDs.

Faced with the potential threat of overpressure, fragmentation, impact and heat, EOD technicians wear appropriately engineered protective clothing to minimise these risks associated with an IED blast. Consequently the protective clothing is extremely heavy (>30 kg) and provides a high level of thermal insulation. Combined, these attributes create a situation that is conducive to accelerated internal heat generation. This combined with a microenvironment that minimises the body’s natural capability to dissipate heat, subsequently compromises the EOD technician’s ability to maintain thermoregulatory balance. Once compromised this predisposes the EOD technician to an increased risk of heat strain and potential heat illness [2], [3]. In order to minimise this risk and the potential threat of the IED itself, EOD technicians often utilise robots to assist. However, in certain scenarios robots cannot be deployed.

The scenarios encountered by an EOD technician vary not only in their geographical location, from urban landscapes to arid desert or dense jungles, but also in the intensity with which they are undertaken. The standard practice of EOD involves periods of searching for the target, before undertaking shorter period(s) of activity in close proximity to the explosive device. In an urban setting, for example, the total search time can vary substantially, from a target that is identified prior to the arrival of the EOD technician to that of a non-identified target in a large scale building. It is these combinations of uncertainty that surrounds each unique scenario, which makes the development of appropriate tolerance times a necessity for the health and safety of the EOD technician.

The role of military and non-military protective clothing in the development of heat strain has been evaluated extensively as highlighted by recent reviews [4], [5]. Tolerance times have also been developed by many military organisations for differing levels of battle dress, from standard camouflage uniform through to the highest level of chemical, biological, radiological and nuclear protection [6], [7]. However, the combination of extreme weight and thermal insulation, as produced by the EOD protective clothing, has to date not been appropriately addressed.

Field observational studies focusing on small groups of bomb technicians have highlighted the potential for heat strain, with rapid increases in core temperature and near maximal heart rates occurring in relatively short time periods [2], [3]. However, these investigations were performed in variable environmental conditions on a small number of participants [2], [3] and a systematic investigation of the influence of environmental conditions and work intensity has yet to be conducted. Therefore the aim of the present study was to evaluate the physiological tolerance times while wearing EOD protective clothing in simulated environmental extremes across a range of differing work intensities.

## Materials and Methods

### Ethics Statement

The procedures carried out in this study were approved by the University Human Research Ethics Committee (#1000001160) and participants were informed of the procedures and had any questions answered to their satisfaction prior to giving their written and oral consent to participate. The study conformed to the current Declaration of Helsinki guidelines.

### Participants

Eight healthy physically active, but not heat-acclimated, males recruited from the University staff and student population volunteered to participate in this study [age, mean ± standard deviation (SD), 25±6 years, height 180±7 cm, body mass 79±9 kg, sum of eight skinfolds 76±15 mm, body surface area 1.99±0.1 m^2^, V˙O_2max_ 57±6 ml.kg.min^−1^].

### Experimental Protocol

Participants were required to attend the laboratory on four occasions, separated by a minimum of seven days. The first session involved aerobic capacity testing (V˙O_2max_), body composition acquisition and a familiarisation with the EOD protective clothing and testing procedures. The remaining three laboratory visits involved the participant walking on a treadmill, while wearing the EOD protective clothing, in an environmental chamber set to a Wet Bulb Globe Temperature (WBGT) of 21, 30 or 37°C. During each of these laboratory visits the participant completed three treadmill-walking trials of 2.5, 4 and 5.5 km⋅h^−1^ with a 1% grade. The order of the testing, for both the walking speed and the temperature, was randomised using a random number generator in a controlled crossover design.

### Environmental Conditions

All trials were completed in an environmental chamber (4×3×2.5 m; length, width, height respectively) with 4.7 km⋅h^−1^ simulated wind speed and a radiant heat load (two radiant heaters positioned 0.8–1.8 m from the participant). The 21, 30, and 37°C WBGT conditions were obtained by the following ambient temperatures and relative humidity’s: 24°C, 50%; 32°C, 60%; and 48°C, 20%; respectively. These conditions were also monitored independently of the environmental chamber’s controls (Quest Temp, Airmet, Australia).

### EOD Protective Clothing

During each trial participants wore the Med-Eng**™** EOD9 suit and helmet (Allen Vanguard, Ogdensburg, New York, USA). The suit consisted of a jacket, trousers, groin protection and a helmet (33.4 kg). Participants’ base ensemble consisted of a t-shirt, shorts, socks and underwear. Athletic shoes with a soft rubber sole were also worn during testing. These base ensemble requirements are standardised in accordance with American Society for Testing and Materials (ASTM) F2668-07 [8].

### Aerobic Capacity, Maximum Heart Rate, Body Composition and Familiarisation

V˙O_2max_ and maximum heart rate were determined as per standard laboratory procedure [9]. Following a warm up period, participants determined a comfortable running speed for use during the test. Participants then donned the expired gas analysis equipment (Moxus, AEI Technologies, Pennsylvania, USA) and a heart rate monitor (Polar Team^2^, Kempele, Finland) and stood on the treadmill for resting data collection. The test started at a speed of ∼4 km⋅h^−1^ below the participants’ comfortable running speed, with a 1% grade. On every minute, the speed was increased by 1 km⋅h^−1^, until the chosen speed was attained. Thereafter, the grade was increased by 1% every minute until volitional exhaustion was achieved. Standard criteria for the determination of maximal aerobic capacity and maximum heart rate were applied [10].

Skinfold thickness measures were obtained, using Harpenden callipers (John Bull, West Sussex, UK), on all participants at eight sites (biceps, triceps, subscapular, iliac crest, supraspinale, abdomen, front thigh and medial calf). Girth measures were taken at four body sites (upper arm, waist, hip and calf) on all participants using a tape measure (Rotary Measure, Futaba, Japan). These sites were chosen to represent all body segments and were identified in accordance with the International Society for the Advancement of Kinanthropometry (ISAK) standards and measured by an ISAK accredited anthropometrist.

Participants were also provided the opportunity to familiarise to the EOD protective clothing. This involved the participant donning the protective clothing, walking around the laboratory and on the treadmill at the speeds to be utilised for the trials.

### EOD Protective Clothing Trials

The remaining three sessions followed a similar protocol. Prior to arrival the participants were requested to abstain from alcohol, tobacco, caffeine and strenuous exercise, and to consume 45 ml of water per kg of body mass in the 24 hours preceding each session. The participants were also provided an ingestible core temperature sensor (CorTemp, HQ Inc, Palmetto, FL, USA) to swallow the evening prior. This was to allow sufficient time for the sensor to pass from the stomach to the intestines, where the reading of core body temperature is optimal [11]–[13].

Upon arrival for the three EOD protective clothing sessions, participants were asked to collect a mid-stream urine sample that was assessed for specific gravity (USG). Participants’ with a USG value less than 1.020 were classified as euhydrated and those with higher values were provided with an additional 500 ml of water to be consumed prior to the commencement of the walking trials. Nude body mass was measured to the nearest 50 g (Tanita BWB-600, Wedderburn, Australia) and a cannula was inserted in the antecubital fossa for the attainment of venous blood samples. Samples were collected into five ml serum separating vacutainers for the determination of serum osmolality as previously described [14].

Participants then had a chest strap fitted to provide continuous heart rate recordings (Polar Team^2^, Kempele, Finland) and thermocrons (ibutton, OnSolution, Baulkham Hills, Australia) to record skin temperature. The thermocrons were attached to four sites (back of neck, inferior border of right scapula, dorsal right hand and proximal third of right tibia) as per International Standard Organisation 9886 (ISO 9886) [15].

Participants then donned the EOD protective clothing and entered the environmental chamber to commence the trial. During each trial, standard termination criteria were applied in accordance with the ASTM guidelines [8]: (1) core body temperature reaching 39.0°C; (2) 60 minutes of exercise; (3) heart rate >90% of maximum; or (4) fatigue or nausea. Following the attainment of one of the termination criteria, the participant exited the environmental chamber and doffed the EOD protective clothing. Nude body mass was undertaken following towel drying and a venous blood sample drawn for determination of serum osmolality. Participants were then instructed to rest in an air-conditioned laboratory. In the following recovery period participants were provided with food and fluid to a volume equivalent to 125% of the body mass loss in the preceding trial. This was undertaken to ensure recovery of body mass and hydration status prior to commencement of subsequent trials. Core temperature and heart rate were monitored and following their return to baseline levels the participant provided a blood sample, had their nude body mass assessed and commenced donning the EOD protective clothing for the subsequent trial. Three trials were conducted in this manner per testing session. The presentation of the trials (2.5, 4 and 5.5 km⋅h^−1^ with a 1% grade) was randomised using a random numbers generator across the testing sessions.

### Data Analysis

Mean skin temperature (T_sk_) was calculated from the four thermocrons using equation one [12].
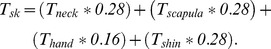
(1)


Mean body temperature (T_wb_) was calculated using equation two proposed by Stolwijk and Hardy [16].
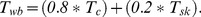
(2)


Physiological Strain Index (PSI) was calculated according to equation three, where T_ci_ (core temperature) and HR_i_ (heart rate) are simultaneous measurements taken at any time during the heat exposure; and T_r0_ and HR_0_ are the resting values. PSI is rated on a scale of 1–10, with five indicating moderate, seven high and nine very high physiological strain [17].
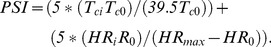
(3)


Metabolic rate was calculated using the load carriage equation of Pandolf [18], where W is the participants body mass (kg), L is the external load comprised of the mass of the EOD9 ensemble, V and G are the treadmill speed (m/s) and gradient (%), and n is the terrain coefficient (1 for treadmill).
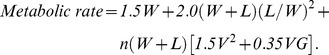
(4)


Body surface area (m^2^) was calculated using the Du Bois equation using height (cm) and mass (kg) [19].
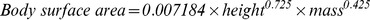
(5)


### Statistical Analysis

All variables were tested for normal distribution with the Shapiro-Wilk test. When the assumption of sphericity was violated, significance was adjusted using the Greenhouse-Geisser method. Tolerance times were analysed using a two-way (environment × speed) repeated measures analyses of variance (ANOVA) with a Bonferroni correction where appropriate. Serum osmolality, body mass loss, sweat loss and the final value recorded for T_c_, T_sk_, T_wb_, HR and PSI were analysed using the same method. All statistical analyses were performed in SPSS (Statistical Package for the Social Sciences), version 19.0 (SPSS Inc, Chicago, IL) with the level of significance set at P<0.05.

## Results

### Baseline (Table 1)

Nude body mass, mean body temperature, heart rate and serum osmolality were similar at the start of all trials. Where multiple trials were performed on the same day the mean duration of rest was 65±13 (range: 43–98) mins.

**Table 1:** Baseline physiological and hydration indices.

Speed(km⋅h^−1^)	HR(bpm)	T_wb_(°C)	Serum Osmolality(mOsmol/kg)	Body Mass(kg)
**2.5**	97±3.6	36.5±0.5	294±1	77.3±2.4
**4**	99±3.1	36.4±0.4	295±1	77.1±2.3
**5.5**	100±2.8	36.5±0.4	294±1	77.6±2.4

Values are means ± SEM. HR, heart rate; bpm, beats per minute; T_wb_, whole body temperature.

### Tolerance Times (Table 2)

Highly significant main effects were observed for environmental condition and speed (P<0.001). No significant (P = 0.166) environment by speed interaction was observed. Post hoc analysis showed tolerance times in WBGT21 were significantly longer than WBGT30 and WBGT37 (P<0.05). In addition, the tolerance times in WBGT30 were longer than WBGT37 (P<0.05). In relation to speed, 2.5 km⋅h^−1^ trials lasted significantly longer than 4 km⋅h^−1^ and 5.5 km⋅h^−1^ (P<0.05). Similarly, 4 km⋅h^−1^ lasted significantly longer than 5.5 km⋅h^−1^ (p<0.05).

### Core Temperature (Table 3)

No significant difference in T_c_ was observed for the main effects of environmental condition (P = 0.108) or speeds (P = 0.117) at the end of the trials. Although a significant environment by speed interaction was observed (P = 0.029), no post-hoc differences existed.

### Skin Temperature (Table 3)

T_sk_ differed significantly between environmental condition (P<0.001) and speed (P = 0.035) at the end of the trials. Post hoc analysis indicated that T_sk_ during the WBGT21 trials were lower compared to both WBGT30 and WBGT37 (P<0.05). However, no difference between WBGT30 and WBGT37 were observed. Using a Bonferroni correction no differences in speeds were evident. No significant environment by speed interaction was observed (P = 0.263).

### Mean Body Temperature (Table 3)

A significant main effect was also observed in T_wb_ in both environmental condition (P = 0.029) and speed (P = 0.037) at the end of the trials. Post hoc analysis indicated that T_wb_ during the WBGT37 trials was greater than those recorded at WBGT21 (P<0.05). However, no differences between WBGT30 and WBGT37 or between WBGT21 and WBGT30 were observed. T_wb_ was also higher at 4 compared to 5.5 km⋅h^−1^ (P<0.05). No differences in T_wb_ were observed between 2.5 and 4 km⋅h^−1^ or 2.5 and 5.5 km⋅h^−1^. A significant environment by speed interaction was observed (P = 0.022). Significant post-hoc comparisons are indicated in Table 3.

### Heart Rate (Table 3)

A significant main effect was also observed in HR in both environmental condition (P = 0.023) and speed (P<0.001) at the end of the trials. Individuals HR at the end of the 2.5 km⋅h^−1^ were lower compared to their HR after the 5.5 and 4 km⋅h^−1^ trials (P<0.05). No differences in HR were observed between the 4 and 5.5 km⋅h^−1^ trials. No post-hoc differences in environmental conditions were evident using the Bonferroni correction. A significant environment by speed interaction was observed (P = 0.046) with post-hoc comparisons indicated in Table 3.

### Body Mass (Table 3)

No significant difference in environmental condition was observed in percentage body mass lost during the trial (P = 0.129). There was however a significant main effect observed between the different speeds (P<0.001). Participants lost a higher percentage of body mass after both the 2.5 and 4 km⋅h^−1^ trials compared to the 5.5 km⋅h^−1^ trials (P<0.05). No differences were observed between the 2.5 and 4 km⋅h^−1^ trials. No significant environment by speed interaction was observed (P = 0.176).

### Sweat Rate (Figure 1)

No significant differences in environmental condition (P = 0.696) or speed (P = 0.161) were observed in sweat rate. Similarly, no significant environment by speed interaction was observed (P = 0.363).

### Physiological Strain Index (Table 3)

The level of physiological strain at the end of the trials was similar across the various environmental conditions (P = 0.060) and speeds (P = 0.054). No significant environment by speed interaction was observed (P = 0.085).

## Discussion

This study provides the first systematic investigation of the effect of work intensity and environmental conditions on physiological strain encountered while wearing EOD protective clothing. The major finding of this study was that participants recorded moderate-high levels of physiological strain but did not experience excessive maximal core, skin or whole body temperature. This level of physiological strain resulted predominantly from participants ceasing trials due to the attainment of the 90% of their maximal heart rate termination criteria.

**Figure 2:**
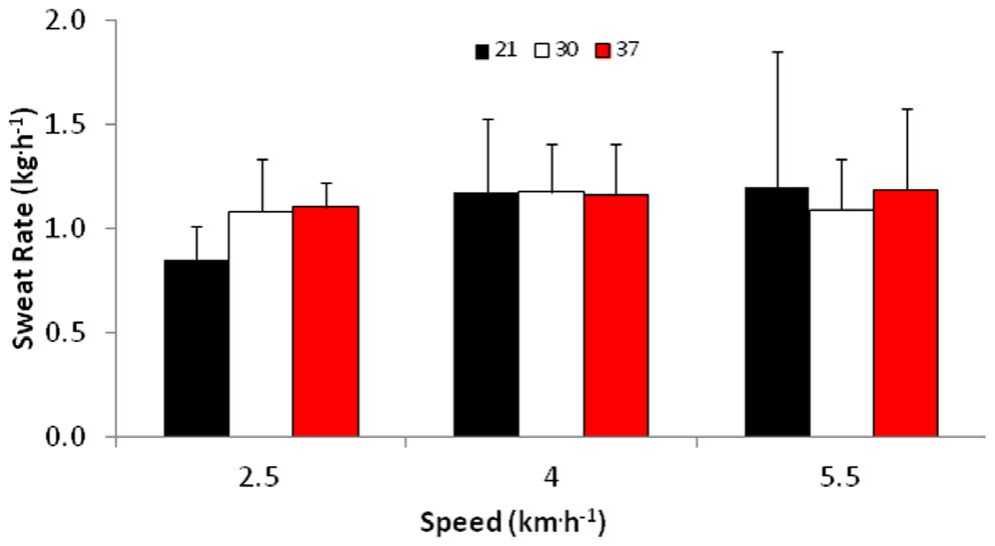
Tolerance time for each environmental condition expressed as a function of individual metabolic rate per body surface area.

Tolerance times achieved were, not surprisingly, significantly reduced in the higher WBGT environments and when the work intensity was increased. With heavy work intensities (>250 W^.^m^−2^), environmental conditions have been shown to have a reduced influence on tolerance time achieved in protective clothing [20]. This results from the delay in sweat evaporation as it moves through the various layers of protective clothing [4]. While there was no significant interaction effect between environments and work intensities in the current study, potentially due to the maximum 60 minute duration creating an arbitrary ceiling at the lower work intensities and environments, the tolerance times achieved at the highest work intensity differed little between the WBGT30 and 37**°**C environments (Table 2, Figure 2).

**Table 2:** Tolerance time (mean, range) and termination criteria for each participant (n = 8) across the different environmental conditions and work rates.

WBGT(°C)	Speed(km⋅h^−1^)	Tolerance Time(min)	HR(>90% max)	Tc(>39°C)	Fatigueor Nausea	Duration( = 60 mins)
21	2.5	57.7 (41.5–60)	1			7
	4	49.6 (27–60)	4		1	3
	5.5	27.6 (14.5–45.5)	8			
30	2.5	52.6 (41.5–60)	2	1	1	4
	4	38.4 (24–55)	7		1	
	5.5	20.3 (10–32.5)	8			
37	2.5	41.0 (27–60)	5		2	1
	4	32.3 (16–50)	7		1	
	5.5	19.1 (10–30.5)	8			

WBGT, wet bulb globe temperature; HR, heart rate; Tc, core temperature.

Almost 70% of the trials (50/72) were ceased due to the attainment of the heart rate termination criteria. Therefore, when we compare the tolerance times at the same work intensities (Table 2, Figure 2), we are able to determine the added stress placed on the heart due to the environmental conditions. The added thermal burden necessitates an elevated cardiac output, represented in the current study by an elevated heart rate response. This accounted for an eight minute or 17% decrease in tolerance time between the WBGT30 and 37°C environments (Table 2); in an attempt to decrease the elevated rate of internal heat storage by increasing skin blood flow and subsequent evaporative heat loss [21]. Previous field investigations of heat strain in EOD technicians have produced similar responses with respect to near maximal heart rates occurring by the completion of the simulated work tasks [2], [3]. These studies were conducted in WBGT 25–34°C environmental conditions, utilising the same EOD protective clothing ensemble.

**Figure 1:**
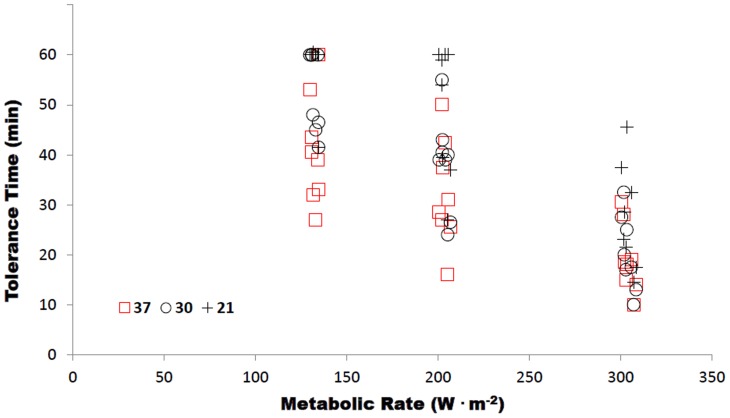
Sweat Rate (Body mass change as a function of tolerance time). Values are mean ± SD.

The increased heart rate, reflecting not only the intensity of the work but an increase in skin blood flow [21], facilitates evaporative heat loss as sweat is phase changed on the surface of the skin. Sweat rates were on average 1.11 kg^.^h^−1^ which is typical of moderate exercise performed in a hot environment [22] (Figure 1). However, values did range up to 2.7 kg^.^h^−1^, which are higher than normally reported by athletes’ engaged in intermittent sporting activities, but have been observed in heat-adapted and endurance-trained athletes [22]. It is worth noting that information regarding the evaporative capacity of this EOD ensemble is limited and further research is required to address this gap in the literature.

The elevated heart and sweat rates are markers of heat loss mechanisms as the body attempts to maintain a thermal balance. Core temperature is the regulated variable and in the current study, given the tolerance times achieved, this did not have an opportunity to reach an excessive level despite an uncompensable heat stress environment. Core, skin and whole body temperature, attained at the cessation of trials (Table 3), were all representative of values obtained previously with an identical EOD protective clothing ensemble in the field [2], [3] or with lighter ensembles utilising intermittent activity of a similar duration in previous laboratory studies [23], [24]. Maximal core temperatures achieved were not significantly different across the work intensities and environmental conditions investigated (Table 3) indicating that the rate of heat storage was greater in the higher intensity and WBGT trials as the time spent in these was significantly less by the participants.

**Table 3:** Physiological data at the cessation of each trial.

WBGT(°C)	Speed(km⋅h^−1^)	HR(bpm)	T_c_(°C)	T_sk_(°C)	T_wb_(°C)	PSI	Body MassLoss (%)
**21**	**2.5**	141.7 (109–174)^b^	37.8 (37.4–38.5)	36.3 (35.8–36.8)	37.5 (37.1–38.1)	4.3 (2.1–6.6)	1.2 (1.0–1.4)
	**4**	162.6 (125–178)	38.1 (37.5–38.6)	36.5 (35.9–37.4)	37.8 (37.2–38.4)	5.9 (3.7–7.4)	1.5 (0.7–2.4)
	**5.5**	173.3 (161–185)	38.0 (37.5–38.8)	36.4 (35.0–37.3)	37.7 (37.0–38.3)	6.2 (4.1–7.9)	1.0 (0.4–2.5)
**30**	**2.5**	156.1 (139–179)^b^^,^ c	38.4 (37.9–39.0)	37.2 (36.8–37.4)	38.2 (37.7–38.7)	5.9 (4.5–7.0)	1.5 (1.0–2.3)
	**4**	169.6 (156–190)	38.3 (37.8–38.7)	37.4 (36.9–38.9)	38.1 (37.7–38.6)	6.6 (5.7–7.5)	1.2 (0.6–2.0)
	**5.5**	172.5 (166–181)	38.1 (37.6–38.8)	36.8 (35.6–37.7)	37.8 (37.2–38.5)	6.3 (5.3–8.3)	0.7 (0.4–1.1)
**37**	**2.5**	161.9 (121–177)	38.2 (37.8–38.8)	37.9 (37.0–38.8)	38.2 (37.8–38.7)^a^	6.0 (1.8–7.6)	1.2 (0.7–1.8)
	**4**	172.3 (165–178)	38.1 (37.5–38.8)	38.0 (36.5–38.7)	38.1 (37.5–38.8)	6.7 (5.3–8.4)	1.1 (0.6–2.1)
	**5.5**	174.6 (167–186)	37.7 (37.1–38.3)	37.6 (35.8–38.9)	37.7 (37.1–38.3)	5.9 (4.8–7.5)	0.8 (0.4–1.3)

Values are mean (range). WBGT, wet bulb globe temperature; HR, heart rate; bpm, beats per minute; T_c_, core temperature; T_sk_, skin temperature; T_wb_, whole body temperature; PSI, physiological strain index.

a significantly different to the same speed at WBGT 21°C (P<0.05).

b significantly different to 5.5 km⋅h^−1^ at the same environmental condition (P<0.05).

c significantly different to 4 km⋅h^−1^ at the same environmental condition (P<0.05).

The current investigation suggests that fatigue and work tolerance when wearing EOD personal protective equipment is based on cardiovascular rather than thermal strain. Regardless of the environmental conditions, all of the trials in the highest metabolic work rate (5.5 km⋅h^−1^) were terminated due to excessive heart rate and no individual completed the 60 min of walking at this intensity (Table 2). Similar findings have also been reported in a recent review of encapsulated protective clothing by McLellan and colleagues [4].

The current study involved the undertaking of multiple trials, in the same environmental conditions, within the same day. This experimental design was planned in order to mimic the activities of the EOD technician in the field. These activities often involve extended searching for the target, however, once it is identified, the technician may make multiple trips to the target in order to obtain x-rays of the target, this is then followed by disarmament or disablement of the device, then a final clearance of the site. These multiple trips are interspersed by periods when the EOD technician is at a safe distance from the device and is able to remove components of the protective clothing. Undertaking multiple trials on the same day, a protocol previously employed in EOD investigations [24], may have had the unintended consequence of participants undertaking subsequent trials in a hypohydrated or elevated physiological strain state. Commencing tolerance tests in a hypohydrated state has previously been shown to reduce completion times by up to 20% [25], [26], however, neither pre-trial nude body mass nor serum osmolality were significantly different between trials in the current study (Table 1). Tolerance times would also be reduced if any of the physiological termination criteria were elevated on commencement of subsequent trials, however, there were no statistical differences in pre-trial heart rate or whole body temperature (Table 1). Moreover, Kenefick and colleagues [27] have previously shown that if hydration and body temperatures are allowed to recover then subsequent aerobic activity in the heat is not impaired. In the current study, these markers of hydration status and heat strain were not significantly different, indicating that the rapid rehydration protocols and the rest periods (∼60 min) employed, enabled subsequent trials on the same day not to be detrimentally influenced by the preceding trials. These findings provide an evidence-base for effective rehydration protocols and work-rest periods that would be required if complete physiological recovery was necessary in the field. This recovery time frame is approximately four times that which is normally implemented by EOD technicians, therefore the challenge of future research should be to evaluate the cumulative effect of the uncompensated heat stress and the capability of cooling strategies to mitigate any heat strain developed.

### Conclusions

The study has provided a comprehensive evaluation of the physiological tolerance times while wearing EOD protective clothing. This was undertaken in simulated environmental extremes across a range of differing work intensities using a systematic approach. This study found that participants displayed moderate-high levels of physiological strain but did not experience excessive maximal core, skin or whole body temperatures.

## References

[1] Roeder RA, Schulman CI (2010) An overview of war-related thermal injuries. J Craniofac Surg 21: 971–975. 20613571 10.1097/SCS.0b013e3181e1e802

[2] Stewart IB, Rojek AM, Hunt AP (2011) Heat strain during explosive ordnance disposal. Mil Med 176: 959–963. 21882791 10.7205/milmed-d-11-00052

[3] Stewart IB, Townshend A, Rojek AM, Costello JT (2013) Bomb disposal in the tropics: a cocktail of metabolic and environmental heat. J Ergonom S2: 001.

[4] McLellan TM, Daanen HAM, Cheung SS (2013) Encapsulated environment. Compr Physiol 3: 1363–1391. 23897690 10.1002/cphy.c130002

[5] Larsen B, Netto K, Aisbett B (2011) The effect of body armor on performance, thermal stress, and exertion: a critical review. Mil Med 176: 1265–1273. 22165654 10.7205/milmed-d-10-00470

[6] Montain SJ, Sawka MN, Cadarette BS, Quigley MD, Mckay JM (1994) Physiological tolerance to uncompensable heat-stress - effects of exercise intensity, protective clothing, and climate. J Appl Physiol 77: 216–222. 7961236 10.1152/jappl.1994.77.1.216

[7] White MK, Hodous TK, Vercruyssen M (1991) Effects of thermal environment and chemical protective clothing on work tolerance, physiological-responses, and subjective ratings. Ergonomics 34: 445–457. 1860463 10.1080/00140139108967328

[8] ASTM Standard F2668–07 (2011) Determining the physiological responses of the wearer to protective clothing ensembles. ASTM International, West Conshohocken, PA, DOI: 10.1520/F2668-07, www.astm.org.

[9] Hunt A, Feigl B, Stewart I (2012) The intraocular pressure response to dehydration: a pilot study. Eur J Appl Physiol 112: 1963–1966. 21877145 10.1007/s00421-011-2143-5

[10] Townshend AD, Worringham CJ, Stewart IB (2010) Spontaneous pacing during overground hill running. Med Sci Sports Exerc 42: 160–169. 20010117 10.1249/MSS.0b013e3181af21e2

[11] Byrne C, Lim CL (2007) The ingestible telemetric body core temperature sensor: a review of validity and exercise applications. Br J Sports Med 41: 126–133. 17178778 10.1136/bjsm.2006.026344 PMC2465229

[12] Goodman DA, Kenefick RW, Cadarette BS, Cheuvront SN (2009) Influence of sensor ingestion timing on consistency of temperature measures. Med Sci Sports Exerc 41: 597–602. 19204591 10.1249/MSS.0b013e31818a0eef

[13] Sleivert GG (2007) Using microtechnology to monitor thermal strain and enhance performance in the field. Int J Sports Physiol Perform 2: 98–102. 19255458 10.1123/ijspp.2.1.98

[14] Taylor NA, van den Heuvel AM, Kerry P, McGhee S, Peoples GE, et al (2012) Observations on saliva osmolality during progressive dehydration and partial rehydration. Eur J Appl Physiol 112: 3227–3237. 22230919 10.1007/s00421-011-2299-z

[15] International Organisation for Standardisation (2004) ISO 9886: Ergonomics - Evaluation of thermal strain by physiological measurements. Geneva: International Organisation for Standardisation.

[16] Stolwijk J, Hardy J (1966) Temperature regulation in man–a theoretical study. Pflugers Arch Gesamte Physiol Menschen Tiere 291: 129–162. 10.1007/BF00412787 5234151

[17] Moran DS, Shitzer A, Pandolf KB (1998) A physiological strain index to evaluate heat stress. Am. J. Physiol Regul Integr Comp Physiol 275: R129–134. 10.1152/ajpregu.1998.275.1.R129 9688970

[18] Pandolf KB, Givoni B, Goldman RF (1977) Predicting energy expenditure with loads while standing or walking very slowly. J Appl Physiol 43: 577–581. 908672 10.1152/jappl.1977.43.4.577

[19] Du Bois D, Du Bois EF (1989) A formula to estimate the approximate surface area if height and weight be known. Nutrition 5: 303–311. 2520314

[20] McLellan TM, Jacobs I, Bain JB (1993) Influence of Temperature and Metabolic-Rate on Work Performance with Canadian Forces NBC Clothing. Aviat Space Environ Med 64: 587–594. 8357310

[21] Cheung SS (2010) Advanced environmental exercise physiology: Human Kinetics.

[22] Sawka MN, Burke LM, Eichner ER, Maughan RJ, Montain SJ, et al (2007) Exercise and fluid replacement. Med Sci Sports Exerc 39: 377–390. 17277604 10.1249/mss.0b013e31802ca597

[23] Thake C, Zurawlew M, Price M, Oldroyd M (2009) The effect of heat acclimatisation on thermal strain during Explosives Ordnance Disposal (EOD) related activity in moderate and hot conditions. Proceedings of the 13th International Environmental Ergonomics Conference, Boston, USA.

[24] Frim J, Morris A (1992) Evaluation of personal cooling systems in conjunction with explosive ordnance disposal suits. Ontario: Defence and Civil Institute of Environmental Medicine: 1–64.

[25] Cheung SS, McLellan TM (1998) Heat acclimation, aerobic fitness, and hydration effects on tolerance during uncompensable heat stress. J Appl Physiol 84: 1731–1739. 9572824 10.1152/jappl.1998.84.5.1731

[26] Cheung SS, McLellan TM (1998) Influence of hydration status and fluid replacement on heat tolerance while wearing NBC protective clothing. Eur J Appl Physiol Occup Physiol 77: 139–148. 9459534 10.1007/s004210050312

[27] Kenefick RW, Ely BR, Cheuvront SN, Palombo LJ, Goodman DA, et al (2009) Prior heat stress: effect on subsequent 15-min time trial performance in the heat. Med Sci Sports Exerc 41: 1311–1316. 19461533 10.1249/MSS.0b013e3181988c14

